# Professional Notes

**Published:** 1887-10-29

**Authors:** 


### Professional Notes.

By a Physician.

Skin diseases cannot be said to constitute a department of practice in which the family doctor is seen at his best. A good many practitioners indeed know very little about them. That is to a considerable extent the fault of the medical schools at which they have been educated, and of the curriculum of study through which they have had to pass. To many, skin affections appear to be of an accidental and arbitrary character, acknowledging no general rules, related by no affinities, and incapable of scientific classification. That is a mistake. Here, as elsewhere, there are certain well-known principles, the thorough understanding of which is of the greatest possible service, both in diagnosis and treatment. A few words from the introductory chapter of the fifth edition of Dr. Liveing's book are well worthy of consideration in this connection. "I would point out," says Dr. Liveing, "in the first place, that the diagnosis of a disease, to be of any practical value, includes much more than assigning to it a name; it involves a correct estimate both of its nature and of its affinities. *The chief feature in the great majority of skin affections is an inflammation*, and in this respect they are very much alike, though they may differ altogether in important respects." That is a principle of the utmost importance in practice. Do most of the skin diseases manifest an inflammatory character as their chief feature? Then most of them should have treatment suitable for structures in a state of inflammation. The constant recognition of this single generalisation is at least half the battle in daily practice. By way of illustration, Dr. Liveing continues: "Thus, in almost all inflammations of the skin, we may chance to find red patches, papules, vesicles, blebs, pustules, scales, or crusts; and, therefore, the first point of importance is the recognition of the fact that these varying phenomena of cutaneous inflammation are simply brought about by the anatomical structure of the skin, and are of uncertain value in determining the nature of the inflammation." All these diseases have the one common characteristic of inflammation, although that inflammation is modified by a great variety of circumstances.

---

"For the recognition of disease of the skin," says Hebra, "no other assistance is required than a knowledge of the objective symptoms, which are visible on the surface of the body in each particular case. We do not attach any value whatever either to the history or to the subjective phenomena in investigating a cutaneous affection, for we ought to be guided in this matter only by those symptoms which are appreciable by the sight, the touch, or sometimes the smell. These afford certain and infallible grounds for the establishment of a diagnosis, for they have their origin in the malady itself. They are, so to speak, the alphabet of which the letters are traced on the skin, and our task is but that of deciphering the writing." Experienced persons will at once think of exceptions to this rule: but for ordinary practitioners, whose opportunities of understanding skin diseases are very limited, it is difficult to imagine any single direction which will more surely lead the traveller right. Look, feel, smell,—these are principal avenues of knowledge in skin diseases.

---

In the treatment of skin affections arsenic is very frequently administered, and the views of an experienced practitioner like Dr. Liveing as to its use are of great value. "The best preparation of arsenic is the liquor arsenicalis, or if an acid preparation is required, the liquor arsenici hydrochloratis. These should be administered in some aromatic or bitter infusion three times a day, directly after the three principal meals. In children, liquor arsenicalis may be conveniently given with a little liquor potassæ, syrup and water the essential point in all cases being that it shall be given on a full stomach. . . . In determining the dose of liquor arsenicalis it is well first to consider what it is we want to produce, namely, an active change in the nutrition of the skin. Now arsenic is an excellent tonic for children in very small doses (♏j), and we often use it simply as such in the treatment of scrofula or eczema; but when we have to deal with psoriasis we want to produce a more marked and specific effect, and hence larger doses must be given. It is always best to begin with moderate doses . . . . and then to increase the dose quickly to the full amount. In children over three years of age, from two to three minims, and for adults from five to ten minims three times a day would be a full dose. I have never seen the slightest permanent ill-effect produced by arsenic when properly administered." It is certain that many practitioners fail of success with arsenic by giving too small doses, or by administering the larger doses unskilfully. By such these directions of Dr. Liveing deserve to be carefully considered.

---

"Common Errors in the Treatment of Skin Diseases" was recently the subject of a paper read by Dr. Fox, at a meeting of the Medical Chirurgical Society of Montreal. He said that the great error made by practitioners was the failure to treat the patient; the disease is treated, not the patient. It it most important that the patient have fresh air, wholesome food, and everything else that tends to improve the general health. Special treatment is of no avail otherwise. Attention to diet is essential. There should be a radical change both in the quantity and quality of the food. The majority of patients improve on starvation diet. The fluids should be increased, the solids diminished; eat less, and drink more. Exercise is indispensable where possible. The more complete the change of diet the better. The best therapeutical effects result from a vegetable diet in inflammatory skin affections. A meat diet congests the skin; a vegetable diet relieves the congestion. In winter meat should be restricted; in summer forbidden. Patients should be told what to eat rather than what to avoid. With meals, water should be taken sparingly; between meals, abundantly.

---

Endocarditis is universally understood to accompany attacks of acute rheumatism, and by all careful practitioners is searched for and treated as early as possible after the commencement of the attack. It is not so generally remembered that this formidable complication may be associated with scarlatina, measles, septicæmia, acute osteomyelitis, and small-pox. Dr. Ernest Sansom in the second edition of his book on valvular disease, calls attention to this neglected field of practice, and it may be hoped that practitioners will feel it their duty to exercise greater watchfulness in these directions. Many cases of scarlatina and other fevers result in prolonged and sometimes permanent disability from these causes. Dr. Sansom, among other methods of practice, recommends vigorous exercise and mountain climbing in certain cases of mitral regurgitation, a view which used to be strongly urged upon his students by so eminent an authority as the late Sir Robert Christison. The indiscriminate treatment of heart affections by enforced idleness and indoor life, although often advised by men of the highest eminence in the profession, cannot be too sternly reprehended. In almost every case it does the patient no good at all, and in many instances a very great deal of harm.

